# Serotype Distribution, Antimicrobial Susceptibility, and Multilocus Sequencing Type (MLST) of *Streptococcus pneumoniae* From Adults of Three Hospitals in Shanghai, China

**DOI:** 10.3389/fcimb.2019.00407

**Published:** 2019-11-27

**Authors:** Xin-Xin Li, Shu-Zhen Xiao, Fei-Fei Gu, Sheng-Yuan Zhao, Qing Xie, Zi-Ke Sheng, Yu-Xing Ni, Jie-Ming Qu, Li-Zhong Han

**Affiliations:** ^1^Department of Clinical Microbiology, Ruijin Hospital, Shanghai Jiao Tong University School of Medicine, Shanghai, China; ^2^Usher Institute, University of Edinburgh, Edinburgh, United Kingdom; ^3^Department of Infectious Diseases, Ruijin Hospital, Shanghai Jiao Tong University School of Medicine, Shanghai, China; ^4^Department of Pulmonary Medicine, Ruijin Hospital, Shanghai Jiao Tong University School of Medicine, Shanghai, China

**Keywords:** *Streptococcus pneumoniae*, serotype distribution, antimicrobial susceptibility, molecular epidemiology, pneumococcal pneumonia, pneumococcal conjugate vaccine

## Abstract

**Background:** *Streptococcus pneumoniae*, a main causative agent associated with invasive and non-invasive infection in elderly population, is a major global health problem. After pneumococcal conjugate vaccines (PCV) and pneumococcal polysaccharide vaccines (PPV) were introduced, the distribution of *S. pneumoniae* serotypes has changed. There was currently limited data on epidemiology and status of antimicrobial resistance of *S. pneumoniae* in Shanghai.

**Objective:** To determine the serotype distribution, antimicrobial susceptibility and molecular epidemiology of *S. pneumoniae* isolated from adults in Shanghai.

**Method:** A total of 75 *S. pneumoniae* isolates consecutively collected from 2015 through 2017 were serotyped by conventional multiplex-PCR. The antimicrobial susceptibility was determined by broth microdilution method. The multilocus sequence type (MLST) was performed to estimate the molecular epidemiology.

**Results:** The predominant serotypes among the isolates were 19F (20.00%), 3 (16.00%), 23F (9.33%), 14 (8.00%), and19A (5.33%). The prevalence of pneumococcal strains with serotypes targeted by vaccines PCV7, PCV10, PCV13, and PPV23 was 44, 45.33, 66.67, and 80%, respectively. Penicillin non-susceptible *S. pneumoniae* (PNSSP) accounted for 16% of the isolates examined and resistance to erythromycin, azithromycin, tetracycline, clindamycin, cefaclor and trimethoprim-sulfamethoxazole were found in 92.00, 90.67, 86.67, 81.33, 54.67, and 54.67% of isolates, with most isolates (78.67%) presenting multidrug-resistance. The top three sequence types (STs) were ST271 (17.33%), ST180 (9.33%), and ST81 (8.00%). The international resistance clone complexes Spain^23F^-1 (*n* = 4), Netherland^3^-31 (*n* = 8), and Taiwan^19F^-14 (*n* = 14) were identified.

**Conclusions:** The *S. pneumoniae* isolates showed high genetic diversity in Shanghai and the prevalence of antimicrobial resistance was also high among *S. pneumoniae* isolates, most of which were multidrug-resistant. The spread of international resistance clones might contribute to the increase of resistant isolates. The PPV23 could protect against most pneumococcal capsular serotypes causing infection of adults in Shanghai.

## Introduction

*Streptococcus pneumoniae* is an opportunistic pathogen that causes both invasive pneumococcal diseases (IPD) including meningitis, bacteremia and non-invasive infection diseases such as otitis media, sinusitis, pneumonia (Engholm et al., 2017). The rate of IPD and the case fatality rate increased with increasing age. Although only 27.3% of the cases were in those aged ≥ 65 years, they accounted for 48% of the deaths (Marrie et al., 2018). The burden of non-invasive pneumococcal disease in adults is mainly determined by community-acquired pneumonia. Pneumococcal pneumonia also shows high incidence rates and carries a high mortality risk, especially in the elderly (Drijkoningen and Rohde, 2014). Chronic diseases and immunocompromise (IC), common in elderly population, are considered as two important risk factors in pneumococcal disease (PD) (Gil-Prieto et al., 2016). It has been presented as a significant cause which leads to substantial economic burden in at-risk and high-risk adults (Zhang et al., 2018).

Vaccines targeting pneumococcus are beneficial to the susceptible population. A study in the US reported that use of the pneumococcal conjugate vaccines (PCV) among children since 2000 has dramatically reduced IPD burden among adults (Moore et al., 2015; Pilishvili and Bennett, 2015). Multiple pneumococcal conjugate vaccines (PCV) and pneumococcal polysaccharide vaccines (PPV) have been developed and introduced to numerous counties (Ochoa-Gondar et al., 2015; Akata et al., 2017; Jauneikaite et al., 2017; Lyu et al., 2017). In China, the 7-valent PCV were first licensed in 2008 and PCV13 and PPV23 are currently on the market (Liang et al., 2016). So far, they have not been covered by the official mandatory vaccination system as a secondary category vaccine. The vaccination is given only on voluntary basis at own expense, which maybe the cause of low vaccination rate in targeting population.

Antimicrobial is used as the most common anti-infection therapy and beta-lactam antibiotic is the first choice for the treatment of pneumococcal pneumonia. The increasing MIC value of penicillin worldwide has been observed and high-level cephalosporin-resistance caught most concern (Hakenbeck et al., 2012). On the other hand, high resistance rate (over 90%) of macrolide and clindamycin, tetracycline has been reported frequently in both pediatric and adult patients in mainland of China (Zhao et al., 2017; Wang et al., 2019). The resistance status is an important reference for antimicrobial selection for anti-infection therapy. The antibiotic-resistance varies in different regions so that local drug resistance monitoring is essential to provide evidence for clinical practice.

Surveillance of serotypes distribution and prevalence of drug-resistance strains in general population is critical for development of appropriate prevention and treatment protocol for *S. pneumoniae* infection. Shanghai is one of the most densely-populated municipals with more than two million people aging over 65 years old. Although the serotype distribution of *S. pneumoniae* and the status of antimicrobial resistance in children has been reported in Shanghai, there has been only limited data for adults in this area (Pan et al., 2015). This multicenter study tried to survey the serotype distribution, antimicrobial resistance and molecular epidemiology of *S. pneumoniae* among adults in Shanghai.

## Materials and Methods

### Patients and Clinical Isolates

The study was conducted at Ruijin Hospital, Renji Hospital and Zhongshan Hospital, which are university-affiliated institutions with over 2000 licensed beds. We performed a retrospective lab-based study of adult patients (over 18 years old) with PD between January 2015 and December of 2017. Cases of PD were identified according to the database in the Department of Clinical Microbiology of each hospital. If the strain was separated from aseptic specimens such as blood and cerebrospinal fluid, it was regarded as IPD. If the strain was separated from non-aseptic specimens such as sputum, it was regarded as non-IPD. Seventy-five episodes of PD were enrolled in this study, only the first isolate of the same type of specimen from the same patient was reviewed and recorded. On basis of colony, microscopic morphology and the production of catalase, optochin test (5 μg in a 6mm disk) was carried out to detect the susceptibility of isolates. If the diameter was over 14mm, the isolate was identified as *S. pneumoniae*. If the diameter ranged from 9 to 13mm, bile-solute test was performed to identity the isolate. Meanwhile, isolates enrolled were also identified using matrix-assisted laser desorption ionization-time of flight mass spectrometer (bioMérieux, Marcy-l'Étoile, France).

This study was approved by the Ethics Committee of Ruijin Hospital affiliated with the School of Medicine at Shanghai Jiao Tong University. The Review Board exempted requirement for informed consent because this retrospective study used only the bacterial samples and had no any negative impact on the patients.

### Antimicrobial Susceptibility Tests

The minimum inhibitory concentrations (MICs) were determined by microdilution method according to the CLSI guideline (CLSI, 2015). Twenty antibiotics were tested (μg/mL): penicillin (0.015~16); amoxillin/clavulanic (0.06/0.03~8/4), cefepime (0.03~4), ceftriaxone (0.03~4), erythromycin (0.06~8), clindamycin (0.03~4), azithromycin (0.06~8), tetracycline (0.12~16), meropenem (0.06~8), ertapenem (0.03~4), imipenem (0.015~2), moxifloxacin (0.06~8), trimethoprim/sulfamethoxazole (0.06/1.2~8/152), rifampin (0.03~4), vancomycin (0.12~16), cefaclor (0.25~16), levofloxacin (0.25~16), linezolid (0.12~8), chloramphenicol (0.5~32), cefuroxime (0.12~8). The breakpoints used for interpretation were recommended by CLSI guideline (CLSI, 2019). *S. pneumoniae* ATCC 49619 was used for quality control.

### DNA Preparation

Rapid DNA extraction was performed by boiling 200μl of bacteria suspension of 1.0 MCF for 10 min. Then the suspension was centrifuged at 12,000rpm/min for 10 min. The supernatant was transferred into a second sterile tube and stocked at −20°C until required.

### Serotyping

Serotyping was performed by sequential multiplex-PCR and primers targeting *cpsA* were used as a positive control in each reaction (Pai et al., 2006). Serotype 6A and 6B were distinguished as previously described (Jin et al., 2009). Serotype 2 and 9N/L were identified by PCR according to previous reported (Dias et al., 2007; da Gloria Carvalho et al., 2010). Other non-vaccine serotypes were detected as published by the Centers for Disease Control and Prevention (CDC)^1^. If serotype that could not identified by conventional multiplex-PCR above, the isolate was classified as untypeable. All primer pairs were listed in Table 1. The prevalence of pneumococcal strains with serotypes targeted by current vaccines were calculated by the percentage of the isolates that the vaccines could protect against.

**Table 1:** Primer pairs of some serotypes in *Streptococcus pneumoniae*.

**Serotype**	**Primer sequence (5^**′**^-3^**′**^)**	**Product (bp)**
1	F:CTCTATAGAATGGAGTATATAAACTATGGTTA	280
	R:CCAAAGAAAATACTAACATTATCACAATATTGGC	
2	F: TATCCCAGTTCAATATTTCTCCACTACACC	290
	R: ACACAAAATATAGGCAGAGAGAGACTACT	
3	F:ATGGTGTGATTTCTCCTAGATTGGAAAGTAG	371
	R:CTTCTCCAATTGCTTACCAAGTGCAATAACG	
4	F:CTGTTACTTGTTCTGGACTCTCGATAATTGG	430
	R:GCCCACTCCTGTTAAAATCCTACCCGCATTG	
5	F:ATACCTACACAACTTCTGATTATGCCTTTGTG	362
	R:GCTCGATAAACATAATCAATATTTGAAAAAGTATG	
6A/B	F:AATTTGTATTTTATTCATGCCTATATCTGG	250
	R:TTAGCGGAGATAATTTAAAATGATGACTA	
6C/D	F: CATTTTAGTGAAGTTGGCGGTGGAGTT	727
	R: AGCTTCGAAGCCCATACTCTTCAATTA	
7F	F:CCTACGGGAGGATATAAAATTATTTTTGAG	826
	R:CAAATACACCACTATAGGCTGTTGAGACTAAC	
7C	F:CTATCTCAGTCATCTATTGTTAAAGTTTACGACGGGA	260
	R:GAACATAGATGTTGAGACATCTTTTGTAATTTC	
8	F:GATGCCATGAATCAAGCAGTGGCTATAAATC	294
	R:ATCCTCGTGTATAATTTCAGGTATGCCACC	
9N/L	F: GAACTGAATAAGTCAGATTTAATCAGC	516
	R: ACCAAGATCTGACGGGCTAATCAAT	
9V	F:CTTCGTTAGTTAAAATTCTAAATTTTTCTAAG	753
	R:GTCCCAATACCAGTCCTTGCAACACAAG	
10A	F:GGTGTAGATTTACCATTAGTGTCGGCAGAC	628
	R:GAATTTCTTCTTTAAGATTCGGATATTTCTC	
10F/10C/33C	F:GGAGTTTATCGGTAGTGCTCATTTTAGCA	248
	R:CTAACAAATTCGCAACACGAGGCAACA	
11A	F:GGACATGTTCAGGTGATTTCCCAATATAGTG	463
	R:GATTATGAGTGTAATTTATTCCAACTTCTCCC	
12F	F:GCAACAAACGGCGTGAAAGTAGTTG	376
	R:CAAGATGAATATCACTACCAATAACAAAAC	
13	F:TACTAAGGTAATCTCTGGAAATCGAAAGG	655
	R:CTCATGCATTTTATTAACCGCTTTTTGTTC	
14	F:CTTGGCGCAGGTGTCAGAATTCCCTCTAC	208
	R:GCCAAAATACTGACAAAGCTAGAATATAGCC	
15A	F:ATTAGTACAGCTGCTGGAATATCTCTTC	436
	R:GATCTAGTGAACGTACTATTCCAAAC	
15B/C	F:TTGGAATTTTTTAATTAGTGGCTTACCTA	496
	R:CATCCGCTTATTAATTGAAGTAATCTGAACC	
16F	F:CTGTTCAGATAGGCCATTTACAGCTTTAAATC	988
	R:CATTCCTTTTGTATATAGTGCTAGTTCATCC	
17F	F:TTCGTGATGATAATTCCAATGATCAAACAAGAG	693
	R:GATGTAACAAATTTGTAGCGACTAAGGTCTGC	
18	F:CTTAATAGCTCTCATTATTCTTTTTTTAAGCC	573
	R:TTATCTGTAAACCATATCAGCATCTGAAAC	
19A	F:GAGAGATTCATAATCTTGCACTTAGCCA	566
	R:CATAATAGCTACAAATGACTCATCGCC	
19F	F:GTTAAGATTGCTGATCGATTAATTGATATCC	304
	R:GTAATATGTCTTTAGGGCGTTTATGGCGATAG	
20	F:GAGCAAGAGTTTTTCACCTGACAGCGAGAAG	514
	R:CTAAATTCCTGTAATTTAGCTAAAACTCTTATC	
21	F:CTATGGTTATTTCAACTCAATCGTCACC	192
	R:GGCAAACTCAGACATAGTATAGCATAG	
22F	F:GAGTATAGCCAGATTATGGCAGTTTTATTGTC	643
	R:CTCCAGCACTTGCGCTGGAAACAACAGACAAC	
23A	F:TATTCTAGCAAGTGACGAAGATGCG	722
	R:CCAACATGCTTAAAAACGCTGCTTTAC	
23B	F:CCACAATTAGCGCTATATTCATTCAATCG	199
	R:GTCCACGCTGAATAAAATGAAGCTCCG	
23F	F:GTAACAGTTGCTGTAGAGGGAATTGGCTTTTC	384
	R:CACAACACCTAACACTCGATGGCTATATGATTC	
24F/A/B	F:GCTCCCTGCTATTGTAATCTTTAAAGAG	99
	R:GTGTCTTTTATTGACTTTATCATAGGTCGG	
31	F:GGAAGTTTTCAAGGATATGATAGTGGTGGTGC	701
	R:CCGAATAATATATTCAATATATTCCTACTC	
33F	F:GAAGGCAATCAATGTGATTGTGTCGCG	338
	R:CTTCAAAATGAAGATTATAGTACCCTTCTAC	
34	F:GCTTTTGTAAGAGGAGATTATTTTCACCCAAC	408
	R:CAATCCGACTAAGTCTTCAGTAAAAAACTTTAC	
35A/C/42	F:ATTACGACTCCTTATGTGACGCGCATA	280
	R:CCAATCCCAAGATATATGCAACTAGGTT	
35B	F:GATAAGTCTGTTGTGGAGACTTAAAAAGAATG	677
	R:CTTTCCAGATAATTACAGGTATTCCTGAAGCAAG	
35F	F:GAACATAGTCGCTATTGTATTTTATTTAAAGCAA	517
	R:GACTAGGAGCATTATTCCTAGAGCGAGTAAACC	
38/25F/A	F:CGTTCTTTTATCTCACTGTATAGTATCTTTATG	574
	R:ATGTTTGAATTAAAGCTAACGTAACAATCC	
39	F:TCATTGTATTAACCCTATGCTTTATTGGTG	98
	R:GAGTATCTCCATTGTATTGAAATCTACCAA	
cpsA	F:GCAGTACAGCAGTTTGTTGGACTGACC	160
	R:GAATATTTTCATTATCAGTCCCAGTC	

### Multilocus Sequence Typing (MLST)

Seven housekeeping genes (*aroE, gdh, gki, recP, spi, xpt*, and *ddl*) were amplified, sequenced, and analyzed. Alleles and sequence types (STs) were determined according to the PubMLST database (https://pubmlst.org/spneumoniae/). Sequence and STs that could not be found in the database were submitted to the curator of the database. The clustering of related STs was analyzed by eBURST Version 3.0.

### Statistical Analysis

Data in this study were analyzed by SAS 8.2 (SAS Institute Inc., Cary, NC, USA). For categorical variables, such as rates of antimicrobial resistance and serotype, the chiq-square test or Fisher test was used to compare the disparity between different groups. *P* < 0.05 was considered statistically significant.

## Results

### Clinical Data

**Figure 1:**
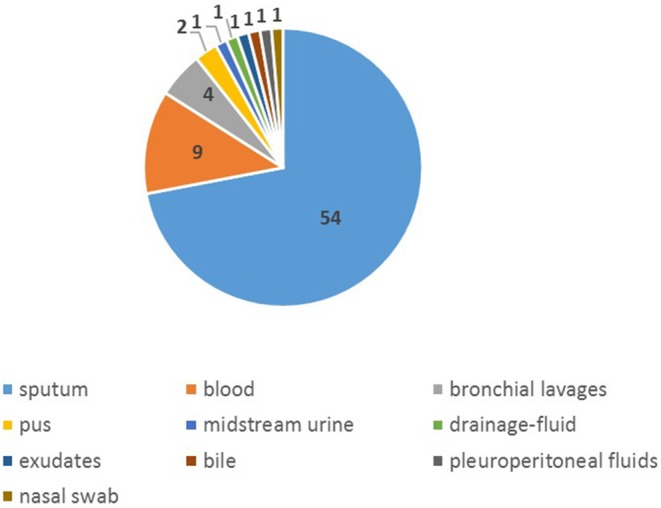
Specimen types of 75 pneumococcal disease cases in Shanghai from 2015 to 2017.

Seventy-five isolates were collected from January 2015 to December of 2017 (Supplementary Table 1), more (65.33%) were from male than female (34.67%). The age of those patients ranged from 28 to 90 years old, of which the mean was 58y and the median was 60 y. The proportion of patients aged over 65 was 37.33%. Among all isolates, 54 were from sputum specimen, 10 from blood and 4 from bronchial lavages and others were from nasal swab, bile, pus, exudates, pleuroperitoneal fluids and drainage-fluid (Figure 1), of which 21.33% were separated from aseptic specimens regarded as invasion, 78.67% from non-aseptic specimens regarded as non-invasion. The proportion of IPD in patients over 65 years old (28.57%) was higher than that in the younger group (17.02%). The common initial diseases in this study was malignant tumor (28.00%) and diseases of respiratory system (16.00%) (Table 2).

**Table 2:** Clinical information of adult patient with PD in Shanghai.

	**19–64y (*n =* 47)**		**>65y (*n =* 28)**		**Total (*n =* 75)**	
	***n***	**Proportion (%)**	***n***	**Proportion (%)**	***n***	**Proportion (%)**
**Gender**						
Female	21	44.68	5	17.86	26	34.67
Male	26	55.32	23	82.14	49	65.33
**PD**						
Invasive	8	17.02	8	28.57	16	21.33
Non-invasive	39	82.98	20	71.43	59	78.67
**Initial diagnosis**						
Respiratory tract infections	4	8.51	3	10.71	7	9.33
Pulmonary diseases^a^	4	8.51	1	3.57	5	6.67
Malignant tumor	11	23.40	10	35.71	21	28.00
Cardio-cerebrovascular disease	5	10.64	2	7.14	7	9.33
Autoimmune disease	3	6.38	0	0.00	3	4.00
Others^b^	3	6.38	7	25.00	10	13.33
Undiagnosed	17	36.17	5	17.86	22	29.33

*Pulmonary diseases^a^, including pulmonary space occupying lesion, pulmonary sarcoidosis, bronchiectasia and chronic obstructive pulmonary disease*.

*Others^b^, including hepatic cirrhosis, jaundice, nephropathy, myopathy, periarthritis, cervical spondylopathy and fever*.

### Serotype Distribution

**Figure 2:**
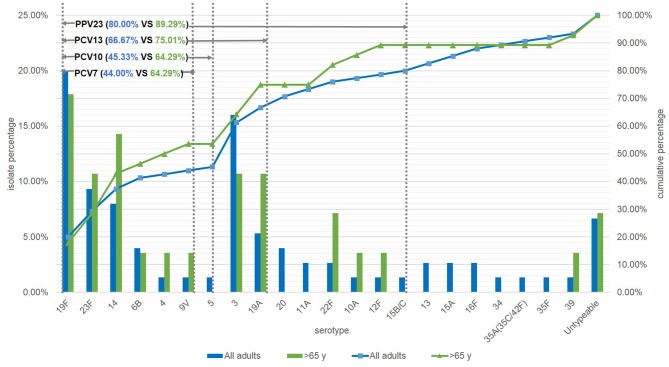
Serotype distribution of *S. pneumoniae* in adults and the coverage of current vaccines in mainland of China. NT, Untypeable by multiplex-PCR.

Among the 75 *S. pneumoniae* isolates, 70 had been properly serotyped. The other 5 isolates were classified as untypeable. Of the serotyped isolates, the most prevalent serotype was 19F (20.00%), followed by 3 (16.00%), 23F (9.33%), 14 (8.00%), and 19A (5.33%). The rates of strains targeted by PCV7, PCV10, PCV13 and PPV23 were 44%, 45.33%, 66.67% and 80%, respectively. The targeted rates in elderly patients (≥65 y) were higher than those in younger patients, which both PCV7 and PCV10 accounted for 64.29% (*P* = 0.0063, *P* = 0.0109), PCV13 for 75.01% (*P* = 0.2373), and PPV23 for 89.29% (*P* = 0.2101) (Figure 2).

### Antimicrobial Susceptibility

The rates of susceptible, intermediate and resistance of *S. pneumoniae* islolates were listed in Table 3. According to the parenteral breakpoint of CLSI guideline, no isolate was resistant to penicillin (MIC ≥ 8 μg/mL), and penicillin-intermediate *S. pneumoniae* (PISP, MIC = 4 μg/mL) accounted for 16.00% of all isolates. For oral breakpoint, the resistant (MIC ≥ 2 μg/mL) and intermediate (MIC = 0.12–1 μg/mL) rates were up to 42.67 and 9.33%, respectively. Most isolates (more than 80%) were resistant to erythromycin, clindamycin, azithromycin, and tetracycline. The resistant rates of cefaclor, cefuroxime, and trimethoprim/sulfamethoxazole were about 50%. There were 14.67 and 17.33% of all isolates resistant to cefepime and ceftriaxone. In penicillin non-susceptible isolates (PNSSP, MIC ≥ 4 μg/mL), the resistant rates of erythromycin, clindamycin, azithromycin, tetracycline and trimethoprim/sulfamethoxazole were 100%, which in PSSP were 90.47, 77.78, 88.89, 84.13, and 46.03%, respectively. Most isolates (over 95.00%) were susceptible to moxifloxacin, levofloxacin, chloramphenicol and all isolates were susceptible to linezolid and vancomycin.

**Table 3:** Antimicrobial susceptibility of 75 *S. pneumoniae* isolates to 20 antimicrobial agents.

**Antibiotics*^a^***	**S (%)**	**I (%)**	**R (%)**	**MIC50**	**MIC90**
PEN (parenteral breakpoint^*^)	84.00	16.00	0.00	0.25	4
PEN (oral breakpoint^*^)	48.00	9.33	42.67	0.25	4
AMC	74.67	6.67	18.67	0.25/0.12	4/8
FEP	65.33	20.00	14.67	0.125	4
CRO	65.33	17.33	17.33	0.125	4
ERY	8.00	0.00	92.00	>8	>8
CLI	18.67	0.00	81.33	>4	>4
AZM	8.00	1.33	90.67	>8	>8
TCY	10.67	2.67	86.67	>16	>16
MEM	52.00	22.67	25.33	0.12	1
ERT	73.33	26.67	0.00	0.25	2
IMP	50.67	40.00	9.33	0.06	0.5
MFX	97.33	1.33	1.33	0.12	0.25
SXT	37.33	8.00	54.67	4	8
RIF	100.00	0.00	0.00	≤ 0.03	0.06
VAN	100.00	0.00	0.00	0.5	0.5
CEC	42.67	2.67	54.67	16	>16
LEV	96.00	0.00	4.00	1	1
LNZ	100.00	0.00	0.00	0.5	1
CHL	93.33	0.00	6.67	2	>4
CXM	49.33	1.33	49.33	0.5	>8

a *PEN, penicillin; AMC, amoxillin/clavulanic; FEP, cefepime; CRO, ceftriaxone; ERY, erythromycin; CLI, clindamycin; AZM, azithromycin; TCY, tetracycline; MEM, meropenem; ERT, ertapenem; IMP, imipenem; MFX, moxifloxacin; SXT, trimethoprim/sulfamethoxazole; RIF, rifampin; VAN, vancomycin; CEC, cefaclor; LEV, levofloxacin; LNZ, linazolid; CHL, chloramphenicol; CXM, cefuroxime*.

* *There was no isolates separated from cerebrospinal fluid so that the all breakpoints were chose for non-meningitis*.

As described in Table 4, two thirds of serotype 19F isolates were non-susceptible to penicillin and more resistant to antibiotics than other serotypes. Moreover, serotype 23F, 14 and 19A showed high resistant rates of cefuroxime. Serotype 3 isolates were more susceptible to these antibiotics than others. The rates of resistance to cefuroxime, trimethoprim/sulfamethoxazole and meropenem in PCV13 targeting isolates were significantly higher than non-PCV13 isolates (the values of χ^2^were 16.67, 4.90, and 7.41 respectively, *P* < 0.05). A similar phenomenon was also observed in ceftriaxone and cefepime in PCV13 group and non-PCV13 group (both χ^2^ = 13.61 *P* < 0.05). There was no significant difference in erythromycin, clindamycin, azithromycin, tetracycline and levofloxacin (*P* > 0.05).

**Table 4:** Resistance rates of *S. pneumoniae* among different serotypes in Shanghai.

**Serotype**	***n***	**PNSSP^a^ (%)**	**CXM^a^ (%)**	**CRO^a^ (%)**	**FEP^a^ (%)**	**MEM^a^ (%)**	**ERY^a^ (%)**	**CLI^a^ (%)**	**AZM^a^ (%)**	**TCY^a^ (%)**	**SXT^a^ (%)**	**LEV^a^ (%)**
19F	15	66.67	100	6.60	20.00	80.00	100	100	100	100	100	13.33
3	12	0.00	0.00	0.00	0.00	0.00	66.67	33.33	66.67	58.33	0.00	0.00
23F	7	0.00	100	0.00	0.00	28.57	100	100	100	100	85.71	0.00
14	6	0.00	83.33	0.00	0.00	16.67	100	100	100	100	50.00	0.00
19A	4	50.00	100.00	0.00	0.00	75.00	100	100	100	100	75.00	0.00
6B	3	0.00	66.67	0.00	0.00	0.00	100	100	100	100	66.67	0.00
20	3	0.00	0.00	0.00	0.00	0.00	100	100	100	100	66.67	33.33
Others	20	0.00	5.00	0.00	0.00	0.00	95.00	80.00	90.00	80.00	35.00	5.00
untypeable	5	0.00	40.00	0.00	0.00	20.00	80.00	60.00	80.00	80.00	60.00	0.00

a *PNSSP, penicillin-non-susceptible S. pneumoniae according to parenteral breakpoint of CLSI; CXM, cefuroxime; CRO, ceftriaxone; FEP, cefepime; MEM, meropenem; ERY, erythromycin; CLI, clindamycin; AZM, azithromycin; TCY, tetracycline; SXT, trimethoprim/sulfamethoxazole; LEV, levofloxacin*.

The resistance patterns were shown in Table 5. Most serotype 19F isolates were PNNSP and were resistance to extensive beta-lactam antibiotics. The most extensive resistance pattern was AMC-FEP-CRO-MEM-IPM-CEC-CXM. Isolates with multidrug-resistance (MDR) accounted for 78.67% (59/75) of all strains. The most common pattern macrolides (ERY and/or AZM)-CLI-TCY existed in 74.67% (56/75) of isolates. The rate of inducible resistance to clindamycin in erythromycin-resistant isolates (*n* = 69) was 10.14% (*n* = 7).

**Table 5:** Resistance pattern of *S. pneumoniae* among different serotypes in Shanghai.

**Serotype**	**Number**	**ST**	**Resistance pattern**
19F	15	271 (*n =* 13), 236, 14665	AMC-CRO-ERY-AZM-CLI-TCY-MEM-IPM-SXT-CEC-CXM (*n =* 2) AMC-FEP-CRO-ERY-AZM-CLI-TCY-MEM-IPM-SXT-CEC-CXM (*n =* 2) AMC-FEP-CRO-ERY-AZM-CLI-TCY-MEM-SXT-CEC-CXM (*n =* 5) AMC-FEP-CRO-ERY-AZM-CLI-TCY-MEM-SXT-CEC-LEV-CXM AMC-FEP-CRO-ERY-AZM-CLI-TCY-SXT-CEC-CXM CRO-ERY-AZM-CLI-TCY-MEM-SXT-CEC-CXM ERY-AZM-CLI-TCY-SXT-CEC-CXM FEP-CRO-ERY-AZM-CLI-TCY-SXT-CEC-CXM FEP-ERY-AZM-CLI-TCY-MEM-MFX-SXT-CEC-LEV-CXM
3	12	180 (*n =* 6), 81 (*n =* 2), 505 (*n =* 2), 875, 4,655	non (*n =* 2) ERY-AZM (*n =* 2) ERY-AZM-CLI ERY-AZM-CLI-TCY (*n =* 2) ERY-AZM-CLI-TCY-CEC-CHL ERY-AZM-TCY (*n =* 2) TCY (*n =* 2)
23F	7	81 (*n =* 4), 180, 1,437, 3,942	ERY-AZM-CLI-TCY-SXT-CEC-CXM (*n =* 4) ERY-AZM-CLI-TCY-CEC-CXM ERY-AZM-CLI-TCY-MEM-IPM-SXT-CEC-CXM ERY-AZM-CLI-TCY-MEM-SXT-CEC-CXM
14	6	876 (*n =* 3), 15, 3,397, 8,561	ERY-AZM-CLI-TCY-CEC-CXM (*n =* 3) ERY-AZM-CLI-TCY-MEM-SXT-CEC-CXM ERY-AZM-CLI-TCY-SXT ERY-AZM-CLI-TCY-SXT-CEC-CXM
19A	4	320 (*n =* 4)	AMC-ERY-AZM-CLI-TCY-IMP-CEC-CXM AMC-ERY-AZM-CLI-TCY-MEM-IPM-SXT-CEC-CXM ERY-AZM-CLI-TCY-MEM-SXT-CEC-CXM AMC-ERY-AZM-CLI-TCY-MEM-IMP-SXT-CEC-CXM
6A/B	3	902, 982, 6,340	ERY-AZM-CLI-TCY-CEC-CXM ERY-AZM-CLI-TCY-SXT ERY-AZM-CLI-TCY-SXT-CEC-CXM
20	3	6,227, 4,745, 9,114	ERY-AZM-CLI-TCY-SXT-CEC-CXM ERY-AZM-CLI-TCY-SXT ERY-AZM-CLI-TCY-CEC
11A	2	99 (*n =* 2)	ERY-AZM-TCY-SXT-LEV ERY-AZM-CLI-TCY-SXT
15A	2	14,607, 11,792	ERY-AZM-CLI ERY-AZM-CLI-TCY-CEC-CXM
16F	2	8,250 (*n =* 2)	ERY-AZM-TCY ERY-AZM-CLI-TCY
22F	2	11,185, 3,465	ERY-AZM-CLI ERY-AZM-CLI-SXT-CHL
4	1		ERY-AZM-CLI-TCY-CHL
9V	1		ERY-AZM-CLI-TCY-SXT
5	1		ERY-AZM-CLI-TCY-CHL
10A	1	4,113	ERY-AZM-CLI-TCY-SXT
12F	1	386	ERY-AZM-CLI-TCY
15B/C	1	14,603	ERY-TCY
13	2	2,754, 11,955	ERY-AZM-CLI-TCY-SXT-CHL ERY-AZM-CLI-TCY
34	1	11,964	ERY-AZM-CLI-TCY-CEC
35A(35C/42F)	1	7,751	ERY-AZM-CLI
35F	1	3,592	TCY
39	1	14,664	ERY-AZM-CLI-TCY-SXT
Untypeable	5	280, 8,356, 1,4,605, 14,606, 1,4697	ERY-AZM-CLI-TCY-SXT-CEC ERY-AZM-CLI-TCY-CEC-CXM ERY-AZM-CLI-TCY-MEM-SXT-CEC-CXM NON ERY-AZM-TCY-SXT

### MLST

Forty-four STs were identified by MLST analysis among 75 isolates. The most prevalent STs were ST271 (*n* = 13, 17.33%), ST180 (*n* = 7, 9.33%), ST81 (*n* = 6, 8.00%). All ST271, ST876, and ST320 isolates were serotyped as 19F, 14, and 19A, respectively. Most of ST180 and ST81 related to serotype 3 and 23F (Table 5). There were seven new ST isolates identified in current study, which had received ST number (ST14603, ST14605, ST14606, ST14607, ST14664, ST14665, and ST14697).

**Figure 3:**
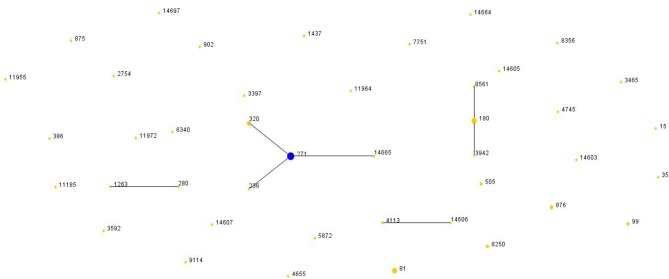
Population snapshot of *S. pneumoniae*. The STs were displayed as a single eBURST diagram by setting the group definition of zero of seven shared alleles. Each number represents one ST and the area of each circle indicates the prevalence of the ST in the MLST data of this study. All 44 STs were shown here in only one group. MLST, Multilocus sequence typing; ST, Sequence type.

There were two clonal complexes (CC) which the primary founders were ST271 with three single-locus variants (SLVs) and ST180 with two SLVs (Figure 3). We defined a group as at least six of seven MLST alleles in common. The ST4113 and ST14606 could be considered to belong to a single clonal complex, so could ST1263 and ST280. And there was no SLV and DLV of ST81.

Compared with Pneumococcal Molecular Epidemiology Network database (PMEN) (at least six of seven alleles in common), there were 34.67% (26/75) of isolates assigned with international resistance clone complexes and their SLVs. The three international resistance clones were Spain^23F^-1 (*n* = 4), Netherland^3^-31 (*n* = 8) and Taiwan^19F^-14 (*n* = 14). Furthermore, Taiwan^19F^-14 clone included 2 STs, the one SLV (ST271, *n* = 13). Netherland^3^-31 clone was also frequently identified in this study including 3 STs, the original Netherland^3^-31 ST180 (*n* = 6) and two SLVs, ST8561 (*n* = 1) and ST3942 (*n* = 1).

## Discussion

*S. pneumoniae* is the principle pathogen that usually caused respiratory infection and invasive infection in children under five years old and senior citizen over 65 years old (Bogaert et al., 2004). Generally, it colonizes the nasopharynx as part of commensal flora in the health person. Once immune system is weaken or ectopic infection occurs, it can caused pneumococcal disease (Bogaert et al., 2004). IPD and pneumococcal pneumonia are common and carry a significant morbidity and mortality. In UK, the incidence of PD (20.58 per 100,000) in those aged > 65 years was significantly higher than that in all aged adults (Chalmers et al., 2016). In this study, chronic diseases and malignant tumor were observed in most patients enrolled, which indicated that chronic diseases and IC were two risk factors of pneumococcal infection. As a previous study in the United States, chronic liver disease and chronic obstructive pulmonary disease (COPD) were the highest risk for IPD. For person with non-IC chronic conditions, IPD risk increased with each additional condition (Baxter et al., 2016). Immunosuppressed patients due to immunosuppressive therapies and immunodeficiency were also at risk of pneumococcal infection. The relative risk (RR) of IPD could be multiplied by 20 to 50 in those with chemotherapy or HIV (Kyaw et al., 2005). Therefore, the prevention of pneumococcal infection in the population is crucial. Vaccines are the most effective tool to reduce the mortality and economic burden caused by pneumococcus (Naucler et al., 2017).

Since 2000, the 7-, 10-, and 13-valent pneumococcal conjugate vaccines (PCV) and 23-valent pneumococcal polysaccharide vaccines were introduced in many countries and reduced PD burden dramatically (Pilishvili and Bennett, 2015). In China, the 7-valent PCV were first licensed in 2008 and PCV13 and PPV23 are currently on the market (Liang et al., 2016). Although PCV13 has been listed in China, it has not been approved for the elderly. The pneumococcal vaccine approved in China for the elderly is PPV23. However, the overall vaccination rate is still unclear in Shanghai. Only a survey reported that in Qingpu district of Shanghai, the vaccination rate of 65–79 years old was 1.6%, the vaccination rate of 80 years old and above was 4.4% in 2014 (A Chinese Expert-workshop Recommendation for Elderly Influenza and Pneumococcal Vaccination, 2018). According to expert recommendation in China, PPV23 is recommended for the elderly aged 60 years and above, and 1 dose for basic inoculation. It is not recommended for the people with normal immune function to be inoculated again. However, if there is a risk factor for severe pneumococcal infection and the first vaccination has been more than 5 years, another vaccination is recommended. People aged 65 and over who have not been vaccinated within 5 years (including those under 65 at the time of the previous vaccination) may be vaccinated again. Due to insufficient safety data for 3 or more doses of PPV23, it is generally not recommended to vaccinate after the second dose (A Chinese Expert-workshop Recommendation for Elderly Influenza and Pneumococcal Vaccination, 2018). It was different from the Centers for Disease Control and Prevention (CDC) of the United States^2^. Although Shanghai has launched a project since 2013 to vaccinate the elderly aged 60 years and above with PPV23 free of charge, the overall vaccination rate in elderly population was still unclear in recent years. As the vaccination project progresses, the vaccination rate in elderly population may have increased. In addition, the distribution of *S. pneumoniae* capsular serotypes in a certain region are associated with local status of vaccines (Akata et al., 2017). So seventy-five *S. pneumoniae* isolates from adults were analyzed to reveal the current prevalence state in Shanghai.

Usually, the introduction of vaccine was followed by a decrease in the rate of vaccine serotype accompanied by an increase in the diversity of non-vaccine serotype (Morales et al., 2018). For example in Japan, PCV13 was licensed in 2013 and the non-PCV13 serotype isolates increased significantly from 2012 to 2014 (Nakano et al., 2016). In a previous study of China from 2011 to 2016, serotype 19F (25.7%), 19A (14.0%), 15 (6.8%), 6B (3.6%), 6A (3.0%), and 17 (2.8%) were prevalent in adults. The prevalence of PCV7- and PCV13-serotypes were 37.5 and 58.3%, respectively (Zhao et al., 2017). The current study showed that the prevalence of serotype 19 group decreased while serotype 3 and 23F increased and the proportion of PCV13-serotypes in Shanghai was higher (66.67%). In patients ≥ 65 years old, the prevalence of PCV7 and PCV10 targeting serotypes were significant higher but those of PCV13 and PPV23 were not obviously different in adult-patients under and over 65y. Although PPV23 covered more serotypes than PCV13, the proportion in current study had no significant difference (*P* = 0.0648 > 0.05) in current study due to the prevalence of serotype 3 and 19A as a report from 2006 to 2016 (Lyu et al., 2017), The current vaccines could still cover most serotypes of the strains causing PD in adults, therefore, the vaccination rate in adult population, especially in the elderly, need to be promoted in the future. In addition, the epidemic data of *S. pneumoniae* from children in Shanghai has been reported previously (Pan et al., 2015). In this surveillance study, we found that the prevalence of 7-, 10-, and 13-valent PCV targeting serotypes were lower than that in children (Pan et al., 2015; Zhao et al., 2019), and it seems that PCV13 was more appropriate for pneumococcal infection protection in children.

Penicillin is normally the first choice for PD treatment. However, as the MIC value of penicillin increases, the breakpoint of CLSI have changed based on the clinical data in 2008. The former breakpoint categorizing susceptible, intermediate and resistant were ≤ 0.06, 0.12–1, and 2 μg/mL, respectively, which are still valid for oral treatment with penicillin and same with the EUCAST criteria. The new breakpoint, ≤ 2, 4, and 8 μg/mL, has established for patients without meningitis but with intravenous treatment (Hakenbeck et al., 2012). In this retrospective study, we found that PNSSP isolates accounted for 16% based on parenteral breakpoint, this rate would have been 52.00% based on oral breakpoint, which was different from that previously reported from Beijing, 8.0% for parenteral breakpoint and 91.6% for oral breakpoint (Lyu et al., 2016). In addition, alterations in the penicillin-binding proteins (PBPs) have been recognized as a major resistance mechanism in *S. pneumoniae* (Hakenbeck et al., 2012). Altered PBP1a, PBP2x and PBP2b are the most important PBPs for β-lactam resistance amongst clinical isolates. It has been found that PBP2b mutation is mainly related to penicillin resistance. And PBP2x mutation can lead to moderate resistance to cephalosporins, while PBP1a mutation can cause high levels of resistance to both penicillin and cephalosporins (Cafini et al., 2006; Izdebski et al., 2008; Davies et al., 2012). In this study, ceftriaxone-resistant isolates (17.33%) were not resistant to penicillin and serotyped as 19F. PBPs mutation of these isolates are required in further research.

The resistant rates of erythromycin, clindamycin, azithromycin and tetracycline were higher than 80%. As reported previously, the resistant rate of erythromycin in China was the highest in Asia (Kim et al., 2012). No matter in children or in adults, resistant rates of *S. pneumoniae* to erythromycin, tetracycline and trimethoprim/sulfamethoxazole were high in China (Yao and Yang, 2008; Zhao et al., 2017; Wang et al., 2019), more serious than that in Europe (Yahiaoui et al., 2016). Levofloxacin and moxifloxacin showed high susceptible rates and no isolates were resistant to vancomycin and linezolid, which was similar with reports in other regions in China (Huang et al., 2015).

*S. pneumoniae* with multidrug-resistance (MDR, resistant to three or more classes of antimicrobial) was widely existed in clinical infection and greatly threaten the treatment of PD. The incidence of MDR in invasive PD was higher than that in non-invasive PD (Wang et al., 2019). In our study, 78.67% of isolates showed MDR. According to a study from Asian Network for Surveillance of Resistant Pathogens (ANSORP), the overall *S.pneumoniae* MDR rate was 59.3%, with the highest MDR rate 83.3% in China (Kim et al., 2012). Vaccine-targeting serotypes demonstrated higher resistance compared with uncovered serotypes. In current study, antimicrobial resistant rate in the most prevalent serotypes (19F, 19A, 14, and 23F) was higher than other serotypes but serotype 3 was much more susceptible. Extensive beta-lactam antibiotic resistance was observed more in 19F. In addition, From 2007 to 2013 in Canada, MDR rate of was only 2.9% and 19F and 19A were also the most common serotype for resistance to more than five antimicrobial classes (Golden et al., 2015).

In previous report of China, CC271 was more common in children, whereas singletons were more prevalent in adults, and serotype 19F and 19A possessed the homogeneous genetic background, together with high resistance to antibiotics (Zhao et al., 2013). The molecular analysis of *S. pneumoniae* in this study showed that ST271, ST180, ST81, ST320, and ST876 were the top five prevalent STs. CC271 was also prevalent in adults, accounted for 25.33% (19/75).The serotype 19F-ST271 was dominating and covered most PNSSP isolates as reported previously (Lyu et al., 2016). The spread of international resistant clones played a predominant role in increasing resistance in Shanghai. The international clone complexes Taiwan^19F^-14, Spain^23F^-1, Spain^6B^-2, and Taiwan^23F^-15 had been reported previously in Shanghai (Yang et al., 2008). In our study, Taiwan^19F^-14 was still predominant in the same region. However, the prevalence of Netherland^3^-31 (ST180) clone was first reported. The international resistance clone Netherland^3^-31 accounted for 10.67% of *S. pneumoniae* from adult, following the Taiwan^19F^-14.

In conclusion, serotype 19F, 3, 23F, 14, 19A targeted by PCV13 and PPV23 were most prevalent in adult patients of Shanghai associated with ST271, ST180, ST81, ST320, and ST876. Taiwan^19F^-14 was predominant of three international resistant clone complexes followed by Netherland^3^-31 (ST180) clone, which was first reported in Shanghai. The incidence of MDR was high and MDR existed in most isolates covered by current vaccines. And the epidemiology of *S. pneumoniae* requires further long-termed investigation on the basis of this study.

## Data Availability Statement

All datasets generated for this study are included in the article/Supplementary Material.

## Ethics Statement

This study was approved by the Ethics Committee at Ruijin Hospital affiliated with the School of Medicine at Shanghai Jiao Tong University. The Review Board exempted requirement for informed consent because this retrospective study used only the bacterial samples and had no any negative impact on the patients.

## Author Contributions

X-XL and S-ZX conceived and designed the experiments, performed the experiments, and wrote the paper. X-XL, S-ZX, F-FG, and S-YZ analyzed the data. QX, Z-KS, Y-XN, J-MQ, and L-ZH contributed reagents, materials, analysis, and tools.

### Conflict of Interest

The authors declare that the research was conducted in the absence of any commercial or financial relationships that could be construed as a potential conflict of interest.

## Footnotes

^1^http://www.cdc.gov/streplab/pcr.html

^2^https://www.cdc.gov/vaccines/vpd/pneumo/hcp/PCV13-adults.html
