# Supplementary material for: Serotype Distribution, Antimicrobial Susceptibility, and Multilocus Sequencing Type (MLST) of Streptococcus pneumoniae From Adults of Three Hospitals in Shanghai, China
Source: Front Cell Infect Microbiol. 2019 Nov 27;9:407. doi: 10.3389/fcimb.2019.00407 (PMC6890718; doi:10.3389/fcimb.2019.00407)
Supplement: Supplementary file 1 [file Table_1.DOCX]

**Supplementary Table 1.** The confidence of MALDI-TOF MS of 75 isolates

| Isolates | The score value of MS |
| --- | --- |
| 74 isolates | 99.9% |
| 1 isolate (NO.1637^1^) | Failed (susceptible to Optochin) |

^1^ Isolate NO.1637, serotyped as 3, was failed to be identified with MALDI-TOF MS because of its abundant mucus. The diameter of the bacteriostatic zone of Optochin was over 14mm.
